# Visual outcomes of the surgical rehabilitative process following open globe injury repair

**DOI:** 10.3389/fopht.2024.1357373

**Published:** 2024-02-14

**Authors:** Richard N. Sather, Sanjana Molleti, Jade Y. Moon, Saliha Chaudhry, Sandra R. Montezuma, Michael Simmons

**Affiliations:** ^1^Department of Ophthalmology and Visual Neurosciences, University of Minnesota, Minneapolis, MN, United States; ^2^Rocky Mountain Retina Consultants, Salt Lake City, UT, United States

**Keywords:** open globe injury (OGI), rehabilitation, trauma, visual outcome, open globe repair

## Abstract

**Background:**

The path of rehabilitation of an eye after open globe injury (OGI) may require multiple additional secondary surgeries after the initial repair. Although much has been studied regarding the outcomes of secondary surgeries after open globe repair, it can be challenging to understand the possible implications of the surgical rehabilitative process. This retrospective study considers the benefits of the required additional secondary surgeries for a consecutive series of OGI patients.

**Methods:**

OGI patients who had at least one additional surgery after the initial open globe repair (OGR) were studied retrospectively. Additional inclusion criteria included: follow up of at least 12 months since the initial injury and at least 3 months since their most recent surgery, and no additional planned interventions. Preoperative visual acuity was compared to final visual acuity. Additionally, the odds of achieving ambulatory vision (≥20/800) and reading vision (≥20/40) were calculated after each indicated consecutive surgery.

**Results:**

A cohort of 74 eyes from 73 patients met our inclusion criteria. These patients underwent a mean of two additional surgeries. The mean logMAR VA improved from 2.3 (HM) at presentation to 1.4 (20/150), or a 9-line Snellen equivalent improvement. Upon reaching their final visit status, 50% of patients had achieved ambulatory vision and 30% of patients had achieved reading vision. The odds of achieving ambulatory vision after completion of all the rehabilitative surgical process compared to the vision prior to the secondary rehabilitative surgery were higher (OR: 19.1, 95% CI: 7.9 – 30.4, p = 0.0008) as were the odds of achieving reading vision (OR: 4.6, 95% CI: 0.2 – 9.0, p = 0.04). With subsequent second, third, and fourth additional surgeries, the odds of achieving either ambulatory or reading vision at the final visit compared to their preoperative visual acuities were not significant (p > 0.05) but the visual acuity continued to trend toward visual improvement.

**Conclusion:**

Approximately 50% of individuals who required additional surgery at UMN achieved ambulatory vision and 30% achieved reading vision. The odds of visual improvement through the surgical rehabilitative process were very high, with the greatest gains generally achieved after the first surgery.

## 1 Introduction

Open globe injuries (OGIs) continue to be one of the leading causes of monocular vision loss in the United States and worldwide (1). OGIs are considered emergencies, and surgery is usually recommended within 12–24 h of the injury (2, 3). The initial surgery includes proper wound closure, addressing prolapsed ocular tissue, and removing blood and tissue that may limit the view for further surgeries (3).

Many OGIs require additional secondary surgeries after the initial repair with the goal of maximizing or restoring vision. The possible interventions may include pars plana vitrectomy (PPV) (4, 5), penetrating keratoplasty (PKP) (6), iris reconstruction, glaucoma surgery (7), cataract surgery (8), and lens placement (9, 10), to name a few. Although much has been studied on the outcomes of anterior and posterior segment surgeries, the entire surgical rehabilitative process after OGI is less well documented. Each surgery poses an increased risk of endophthalmitis, inflammation, or other complications, with the possibility of diminishing returns with additional intervention. Thus, the question of whether to continue to offer surgery to patients with complications of OGI continues to pose a concern for surgeons and their patients (11).

The current literature also describes factors that predict the final visual acuity (VA), including delays in time to surgery, age of patient, preoperative VA, and modality of injury (12–17). Poor prognostic factors including globe rupture, zone III injuries, multimorbidity, history of PKP, retinal detachment (RD), vitreous hemorrhage (VH), and lens dislocation have shown similar results (16, 18, 19). Despite these in-depth findings, there has been increased recognition of the need to evaluate the VA outcomes for patients who undergo secondary surgeries (15).

The Birmingham Eye Trauma Terminology System (BETTS) was proposed by Kuhn et al. in 1996 to help identify all injury types and classify injuries within a comprehensive framework (20). Eye injury terminology is first divided into closed or open globe injuries. Closed globe injuries include either contusion or lamellar laceration. The focus of this study centers around OGIs, which are either rupture or laceration. A laceration is further subdivided into penetrating, perforating, or intraocular foreign body (IOFB). The differences will be addressed in *Methodology*.

The purpose of this study was to evaluate the VA benefits and qualitative results of secondary indicated surgeries (e.g., RD repair and glaucoma surgery), including surgeries for the rehabilitative process (e.g., cataract surgery and cornea transplant) after the initial open globe repair (OGR) to inform future care protocols for ocular trauma patients.

## 2 Methodology

In this retrospective case series, all patients who underwent OGI at the University of Minnesota (UMN)/Minnesota Health System between September 1, 2012 (the date our institution implemented its current electronic medical record system) and October 20, 2022 (the date of IRB submission) were reviewed under our IRB approval STUDY00015830.

The inclusion criteria for this study were as follows: 1) had undergone subsequent surgeries following their OGR; 2) had no further planned surgical intervention; 3) with at least 12 months since their initial injury; and 4) with at least 3 months since their most recent surgery. Patients with non-traumatic globe compromise (e.g., perforation secondary to corneal ulceration) were excluded. Clinical information was gathered using our institution’s Electronic Healthcare Record system. Data collection did not exclude patients based on age, ethnicity, or gender. Patients within our hospital system may opt out of inclusion in retrospective chart reviews at the time of the initial consent for service. All patients who opted out were excluded from this analysis.

### 2.1 Database construction

The REDCap software platform was used to curate the UMN Open Globe Database. An original survey was constructed to facilitate the retrospective collection of all OGIs between the dates mentioned previously. Each patient received a randomized numerical assignment, accompanied by their medical identification number and baseline demographics. Any question addressed in the survey that was not directly found in the patient chart was labeled as “not documented”.

The data entry for each open globe patient included four surveys: 1) initial presentation; 2) initial surgical repair; 3) secondary surgeries; and 4) final outcome. The clinical characteristics of the initial OGI included the Birmingham Eye Trauma Terminology (BETT) classification of the type of globe injury (penetrating, perforating, IOFB, and globe rupture) and the zone of injury (20). Rupture was further divided into non-penetrating keratoplasty (non-PKP) and PKP dehiscence. Additional injury details recorded included the presence of RD, endophthalmitis, VH, relative afferent pupillary defect (rAPD) at presentation, mean laceration length, and the presence of laceration extending 13 mm posterior to the equator. The laceration length was calculated by adding all laceration lengths to determine the longest component. Small segments of branching lacerations were ignored.

### 2.2 Secondary surgeries and final outcome

The types and total number of ophthalmic operations undergone after the initial OGR were recorded. The incidence of proliferative vitreoretinopathy (PVR), RD, eye evisceration/enucleation, and phthisis bulbi was also recorded. The final visual outcome measures included the final Snellen acuity, percentage of those who achieved reading vision (defined as VA ≥20/40), percentage of those who achieved ambulatory vision (defined as ≥20/800), and VA comparison from baseline. A patient was deemed to have reached their final VA if they 1) were at least 12 months out from the initial injury and 2) had no additional surgery planned.

### 2.3 Statistical analysis

Statistical analysis was performed using R v.3.6.3 (R Foundation for Statistical Computing) (21). Mixed effects logistic regression models were used to calculate the odds ratios (ORs) to interpret the likelihood of achieving the visual end points of both reading and ambulatory vision with each secondary surgery and to calculate confidence intervals. Confidence intervals were calculated at the 95th percentile, and statistical significance was set to *p* < 0.05. To calculate the OR, we assumed that, if the patient had not undergone any additional surgery, their final VA would have been equal to their preoperative VA for that surgery. Although we noted the change between the preoperative and postoperative vision for each surgery, we calculated the OR with the patient’s final VA. The preoperative VA for any given surgery was compared to the patient’s final VA (not to their postoperative VA) for that surgery regardless of the number of surgeries a given patient had. We also noted the visual changes that occurred following each surgery to provide a mixed effects regression model, which was used to account for the inclusion of each patient twice in the dataset, once as preoperative self and once as postoperative self. The Snellen VA was converted to a corresponding logMAR scale to reduce erroneous results and misrepresentative statistical analyses (22, 23). To convert low VA reference values [count fingers, hand motion (HM), light perception (LP), and no light perception (NLP)] to logMAR, we utilized the Excel conversion tool produced by Moussa et al. (24).

## 3 Results

### 3.1 Demographic information

A total of 229 patients with OGIs between September 2016 and October 2022 were evaluated. From these patients, a cohort of 74 eyes from 73 patients met the proposed inclusion criteria. The baseline demographic information for our patient cohort is displayed in **Table 1**. The cohort was predominantly men and Caucasian, with a median age of 38 years. The majority of injuries occurred at home (41.1%), at work (26.0%), or on a farm (6.8%). The mechanism of injury was widely dispersed, with the majority coming from ground-level fall/syncope (15.1%), operating a power tool (11.0%), and firearm/firework (11.0%). Approximately a third of the mechanisms did not fall into one of the categories listed.

**Table 1:** Baseline demographic information.

Epidemiological data	Number distribution	Percentage
1. Sex		
Male	63	86.3
Female	10	13.7
2. Age (years)		
Mean ± SD	42.5 ± 22.3	
Median	38	
Range	4–96	
3. Self-identified ethnicity		
Caucasian	63	82.9
Black/African American	8	10.5
Other: American Indian/ Alaska Native/Asian/Latin	5	6.6
4. Eye(s) affected		
OD	32	43.8
OS	40	54.8
OU	1	1.4
5. Injury location		
Home	30	41.1
Work	19	26.0
Farm	5	6.9
School	0	0.0
Other	19	26.0
6. Injury mechanism		
Ground-level fall/syncope	11	15.1
Hammering metal-on-metal	7	9.6
Power tool	8	11.0
Animal	3	4.0
Sport	1	1.4
Assault	6	8.2
Firearm/firework	8	11.0
Sharp stick	4	5.5
Other	25	34.2

OD, oculus dexter; OS, oculus sinister; OU, oculus uterque.

### 3.2 Initial injury and surgical management

The BETT classification system was first utilized at the beginning of each visit. The open globe clinical evaluation is displayed in **Table 2**. There was an almost equal distribution of penetrating (29.2%), IOFB (27.8%), and non-PKP rupture (27.8%) injury types that comprised the majority of our patient cohort. There were only three cases (4.2%) of perforating injury. The injury zone consisted mainly of zone I involvement (63.9%), with a near-equal distribution between zones II and III (27.8% and 20.8%, respectively). The mean Ocular Trauma Score (OTS) was ~2. The mean laceration length was ~9.5 mm, with a median of 7.5 mm. Laceration lengths ranged from 1 to 30 mm posterior to the limbus. The presenting VA ranged from 20/20 to NLP, with an average logMAR of 2.3 (equivalent to HM). The most common clinical findings on initial presentation were RD (30.1%) and VH (54.2%). Approximately 10% of patients had endophthalmitis, rAPD, or a laceration posterior to the equator.

**Table 2:** Clinical characteristics of the initial injury and intraoperative repair.

Preoperative status	Number distribution	Percentage
1. Injury type		
Penetrating	21	29.2
Perforating	3	4.1
IOFB	20	27.8
Rupture (non-PKP)	20	27.8
Rupture (PKP dehiscence)	8	11.1
2. Injury zone		
Zone I	46	63.9
Zone II	20	27.8
Zone III	15	20.8
3. OTS		
Mean ± SD	2.1 ± 1	
Median	2	
Range	1–5	
4. Laceration length (mm)		
Mean ± SD	9.5 ± 7.1	
Median	7.5	
Range	1–30	
5. Presenting VA logMAR (Snellen equivalent)		
Mean ± SD	2.3 ± 0.85 (~HM)	
Median	2.4 (HM)	
Range	0–3.0 (20/20–NLP)	
6. Clinical findings		
Vitreous hemorrhage	39	54.2
RD	22	30.1
Endophthalmitis	7	9.7
rAPD	9	12.3
Laceration posterior to the equator	6	8.7

IOFB, intraocular foreign body; PKP, penetrating keratoplasty; OTS, Ocular Trauma Score; RD, retinal detachment; rAPD, relative afferent pupillary defect; HM, hand motion; NLP, no light perception.

### 3.3 Additional surgeries and final patient outcome

**Figure 1:**
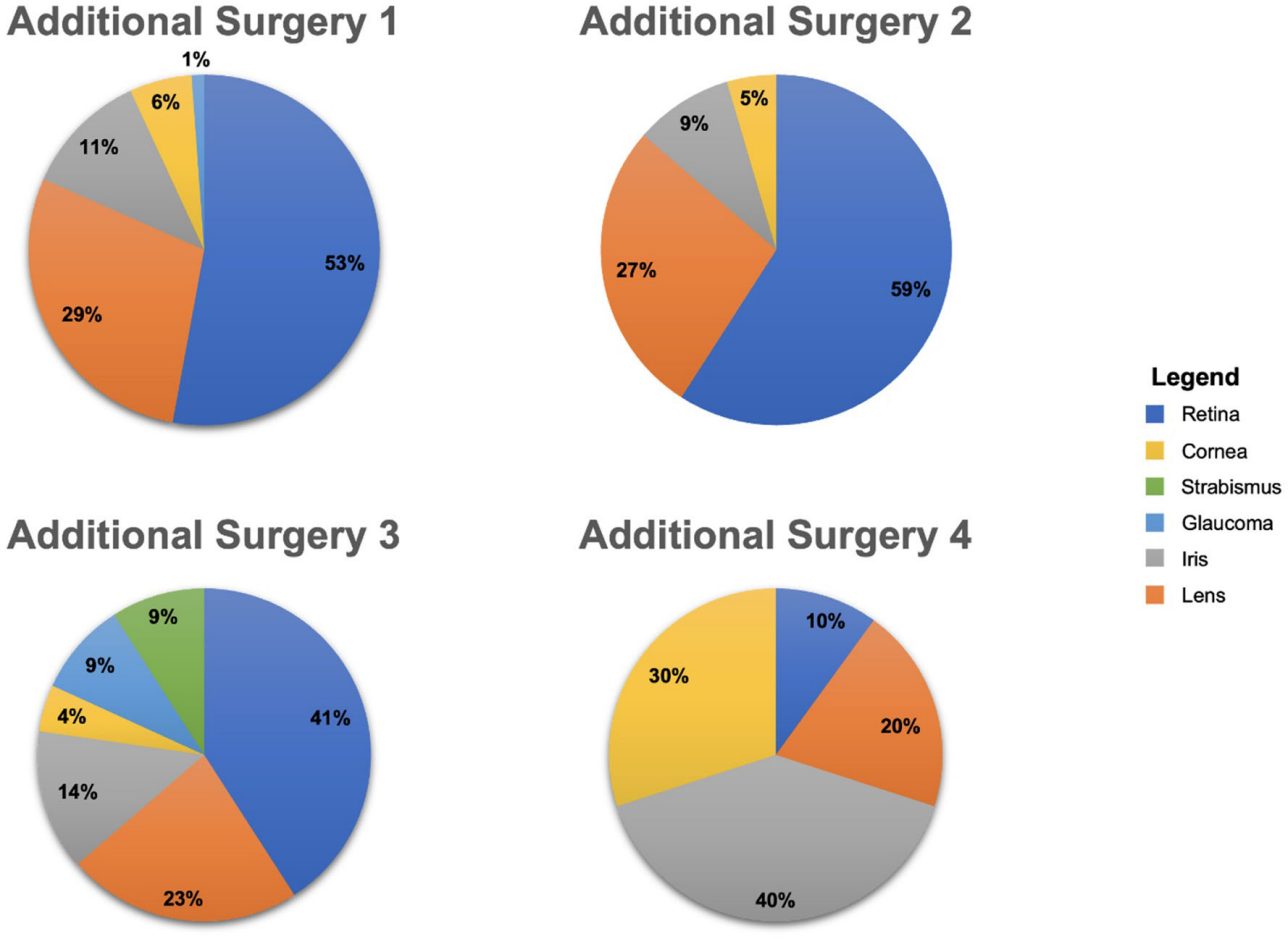
Graphical division of the different types of additional secondary surgeries.

The patients in our cohort underwent a mean of two secondary surgeries after their initial globe repair. **Figure 1** displays the total number of patients who underwent each secondary surgery with attention to each type of secondary surgery. Only two patients had five additional surgeries, and one patient had six. The majority of surgeries were retinal repair (49%), followed by lens management (28%), iris surgery (13%), corneal transplant (6%), and glaucoma surgery (3%). Strabismus surgery represented 1% of the secondary surgeries. The final visual and anatomic outcomes of this cohort are presented in **Table 3**. It should be noted that 12 patients (16.4%) did not get an initial VA. A final mean VA outcome of 1.4, or 20/500, was obtained for our patient cohort. This represents an average of 0.9 logMAR scale, or a nine-line Snellen VA, improvement from the presenting baseline VA. There were 39 patients (~65%) who had a final outcome VA improvement relative to their baseline VA. Subsequently, ~12% of the final VA outcome worsened and ~23% remained unchanged. The 14 patients who had an unchanged final VA included 10 NLP, 3 LP, and 1 HM. Overall, ~50% of our patient cohort achieved ambulatory vision and 30% achieved reading vision.

**Table 3:** Secondary surgeries and final outcome.

Postoperative status	Number distribution	Percentage
1. Secondary surgeries		
Mean ± SD	2.08 ± 1.20	
Median	2	
Range	1–6	
2. Final VA logMAR (Snellen equivalent)		
Mean ± SD	1.4 ± 1.2 (20/500)	
Median	0.88 (20/150)	
Range	0–3 (20/20–NLP)	
Achieved:		
Reading vision ≥20/40	20	28.2
Ambulatory vision ≥20/800	38	53.5
From baseline VA:		
Improved	39	65.0
Worsened	7	11.7
Unchanged	14	23.3
3. Final surgical outcome		
RD incidence	32	44.4
PVR incidence	15	20.8
Retina attached	40	55.6
Retina re-attachment	20	62.5
Phthisis bulbi	2	3.0
Enucleation	13	17.8

VA, visual acuity; RD, retinal detachment; PVR, proliferative vitreoretinopathy.

The retina remained attached for the majority of patients (~55%), while PVR developed in ~20%. Of those subjects who developed RD, re-attachment occurred in 62.5% of patients by their final visit. Overall, ~80% of the patients had their retina attached by the final visit. A total of 13 patients (17.8%) had their eye enucleated as a result of the trauma. Lastly, only two patients (3.0%) developed phthisis bulbi, and both of these were enucleated.

**Figure 2:**
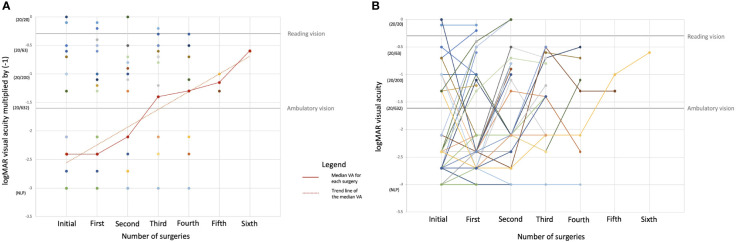
Changes in the preoperative visual acuity (VA) by number of subsequent surgeries. The *y*-axis represents the logMAR VA value multiplied by (−1). **(A)** All VA values with the median VA value for each surgery and the trend line for the median VA value superimposed. **(B)** Changes in the preoperative VA with each subsequent additional surgery by each individual patient. Each of the 73 *colored lines* with its associated *dots* corresponds to individual patients and changes in their VA with each subsequent surgery.

On crude analysis, there was an improvement in the median VA with each subsequent surgery, as demonstrated in **Figure 2A**. This upward trend in VA held true for the majority of individual patients included in our cohort, as demonstrated in **Figure 2B**.

**Table 4** displays the ORs for achieving the visual end points of reading and ambulatory vision at the final visit, with each additional surgery compared to the preoperative VA for that surgery, controlling for OTS. The number of patients who underwent a secondary surgery is indicated by the denominator (i.e., 73 patients underwent the first secondary surgery, 41 patients underwent a second secondary surgery, etc.). The number of patients who needed additional surgery decreased by approximately half after each surgery. The number of patients who achieved ambulatory VA increased to more than double after their first additional surgery and nearly doubled following the second surgery. The number who attained reading VA increased from 2 to 20 and from 2 to 7 patients for secondary surgeries 1 and 2, respectively. It should be noted that the number of patients who achieved ambulatory VA after their third secondary surgery decreased postoperatively; however, the number who achieved reading VA postoperatively increased.

**Table 4:** Patient proportion and odds ratio of obtaining ambulatory and reading visual acuity (VA) with each secondary surgery controlling for the Ocular Trauma Score (OTS).

Secondary surgery no.	Proportion preoperatively	Proportion postoperatively^a^	Odds ratio^b^	95% Confidence interval	*p*-value
Surgery 1:					
Ambulatory	13/73	39/73	19.1	7.9–30.4	0.0008*
Reading	2/73	20/73	4.6	0.2–9.0	0.04*
Surgery 2:					
Ambulatory	12/41	21/41	1.6	−0.1 to 3.3	0.06
Reading	2/41	7/41	3.3	−8.0 to 14.7	0.56
Surgery 3:					
Ambulatory	13/23	12/23	−0.6	−2.5 to 1.4	0.58
Reading	2/23	4/23	−8.2	−72.4 to 55.9	0.80
Surgery 4:					
Ambulatory	5/9	6/9	12.6	−15.0 to 40.2	0.37
Reading^c^	1/9	1/9	–	–	–

*p < 0.05 (statistically significant relationship).

a The postoperative VA for surgery 1 was taken from the same visit as the preoperative VA for surgery 2, and likewise for each surgery.

b The odds ratio was calculated using the final VA. Postoperative proportions meeting the visit thresholds are provided for clarity.

c Statistics unable to perform the fourth surgery (reading) due to a small sample size.

Only two patients required more than four surgeries. The ORs for the achievement of final visual end points were not calculated for these. A single patient who required five secondary surgeries had an IOFB removal with four subsequent retinal surgeries. There was an ~1.2 logMAR improvement between the initial and the final visit (CF → 20/150). Another patient underwent six secondary surgeries due to endophthalmitis complications. This patient underwent three retinal surgeries, one cornea surgery, one lens surgery, and one iris surgery. There was an ~0.8 logMAR improvement (20/800 → 20/60) for this patient at the final outcome. The data of these patients were excluded from **Table 4**, as there were insufficient eyes for statistical analysis.

The odds of achieving ambulatory and reading vision at the final visit after undergoing the first surgery were 19 times greater than without that procedure (OR = 19.1, 95% CI = 7.9–30.4, *p* = 0.0008). With subsequent second, third, and fourth additional surgeries, the odds of achieving ambulatory or reading vision were not significant (*p* > 0.05). The second additional surgery, however, did trend toward significance at *p* = 0.06 for ambulatory vision, with a favorable OR. It should be noted that having a third additional surgery lowered the odds of achieving either ambulatory or reading VA; however, the small sample size likely confounds this measurement. In addition, the small sample size limited the statistical analysis for reading vision after the fourth additional surgery.

## 4 Discussion

In this retrospective case series of patients who underwent additional surgeries after OGR, approximately one-half of our cohort achieved ambulatory vision, while a little over one-fourth achieved reading vision, with a mean of two surgeries by their final visit. For patients and their surgeons contemplating the implications of a recent OGI, these data suggest that, when indicated, the surgical rehabilitative process provided a functionally meaningful visual benefit in our population.

The mean VA on initial presentation in our cohort was logMAR 2.3 (roughly equivalent to HM) and improved to logMAR 1.4 (Snellen equivalent 20/150), which represents a 0.9 logMAR, or a nine-line ETDRS equivalent improvement. The OTS provides predictions of the final VA at 6 months after OGI (20), and these predictions have been validated in other cohorts (25). For our cohort, the mean OTS of approximately 2 on presentation, which corresponds to a 6-month estimated VA follow-up of ≥20/40 (15%), 20/50–20/200 (13%), 19/200–1/200 (18%), HM or LP (26%), and NLP (28%) (26). The patients in our cohort outperformed these predictions, but it should be noted that our cohort was inherently different from that in the OTS: we included only patients who underwent secondary surgeries, which likely biased our cohort to include patients with higher vision potential (i.e., patients who underwent additional surgery did so because their surgeons deemed their vision potential high enough to merit intervention).

In our cohort, the odds of achieving ambulatory and reading vision at the final visit were most favorable with the first additional surgery, and the majority of patients who achieved these end points did so after the first surgery. Following this first surgery, the odds of meeting the VA end points were not significant. A number of factors likely contributed to this. Firstly, the sample size decreased by approximately half with each consecutive surgery, making statistical significance harder to demonstrate. Secondly, the preoperative proportion of patients who had already met a given end point increased with each consecutive surgery so that the difference between preoperative and postoperative VA shrunk as patients underwent additional intervention. Lastly, the number of required surgeries may well reflect the severity of the initial injury: the more surgeries required, the worse the underlying pathology may have been, and the more limited the final visual prognosis.

The division among the different types of secondary surgeries that were performed is included in **Figure 1**. Approximately 50% of our patient cohort underwent at least one secondary retinal surgery. These surgeries were used to manage various posterior pathologies common after OGI, such as RD, VH, PVR, and endophthalmitis. A total of 46 patients (63%) had a PPV performed during the first surgery, and all of those patients had a postoperative VA improvement: 16 eyes improved to ambulatory vision and 11 eyes improved to reading vision. These data align with the findings of other groups whose research supports the importance of vitrectomy after OGR (25).

Approximately 43% of our cohort developed an RD. Given that our cohort was biased toward those who needed surgical intervention after OGI, this percentage is not directly comparable, but is likely consistent with the incidence of RD after OGI of 29.0% reported by the Massachusetts Eye and Ear Infirmary (4) and the Kellogg Eye Center for RD after OGI (27). In our cohort, 55% had their retina attached at the initial presentation. Conversely, 62.5% of patients who experienced RD had reattachment by the final visit. Over 80% of patients had their retina attached at their final visit.

One of the strengths of this study is our approach of calculating the OR for the final VA rather than the postoperative VA because this reflects the clinically relevant question that patients and their surgeons want to know: after sustaining an OGI, if an unknown number of surgeries will be needed to rehabilitate the eye, will taking the next step and undergoing the next indicated surgery be worth it in the end? Although multiple different surgeries may be indicated, using the OR for the final VA allows the ratio to reflect the entire rehabilitation process rather than the specific details of a single indicated surgery. Likewise, the structural outcomes we reported should be interpreted as the outcomes for a cohort who completed a surgical rehabilitative process after OGI.

One limitation of this study is that our categorical outcome markers of ambulatory and reading vision limited recognition of vision gains within these categories (i.e., clinically relevant gains of vision between 20/800 and 20/40 or from 20/40 to 20/20 were not captured in our ORs). Likewise, our results were limited by the extent our assumption is true that the final VA for a given patient would be equal to their preoperative VA if they never underwent any additional intervention. Many surgeries after OGI are performed with the intent of stabilizing the eye (for example in the setting of developing RD or glaucoma), so it is reasonable to assume that the final vision for patients would have been equal to or worse than their preoperative vision if they had not undergone surgery. Lastly, this was a retrospective cohort study from a single institution; thus, our results may not be generalizable to other populations and should be applied in clinical decisions with caution.

## 5 Conclusion

Patients at the University of Minnesota who underwent secondary surgeries after OGR experienced significant gains in vision. The odds of achieving ambulatory vision and reading vision through the surgical rehabilitative process were favorable, and the greatest gains for our cohort were attained through the first secondary surgery. The number of patients needing additional surgery decreased by approximately half with each surgery through the surgical rehabilitative process.

## Data availability statement

The original contributions presented in the study are included in the article/supplementary material. Further inquiries can be directed to the corresponding author.

## Ethics statement

The studies involving humans were approved by University of Minnesota Institutional Review Board. The studies were conducted in accordance with the local legislation and institutional requirements. Written informed consent for participation was not required from the participants or the participants’ legal guardians/next of kin in accordance with the national legislation and institutional requirements.

## Author contributions

RS: Conceptualization, Data curation, Formal analysis, Investigation, Methodology, Writing – original draft, Writing – review & editing. SM: Data curation, Formal analysis, Investigation, Methodology, Writing – review & editing. JM: Data curation, Formal analysis, Investigation, Methodology, Software, Writing – original draft, Writing – review & editing. SC: Data curation, Investigation, Methodology, Writing – review & editing. SM: Formal analysis, Funding acquisition, Investigation, Resources, Supervision, Validation, Writing – review & editing. MS: Conceptualization, Data curation, Formal analysis, Funding acquisition, Investigation, Methodology, Resources, Supervision, Validation, Visualization, Writing – original draft, Writing – review & editing.

## References

[1] Chen A McGwin G Justin GA Woreta FA. The United States eye injury registry: past and future directions. Ophthalmology. (2021) 128:647–8. doi: 10.1016/j.ophtha.2020.11.026 33388159

[2] Makhoul KG Bitar RA Armstrong GW Weinert MC Ivanov A Kahale F. Effect of time to operative repair within twenty-four hours on visual acuity outcomes for open globe injuries. Eye. (2023) 37:2351–5. doi: 10.1038/s41433-022-02350-6 PMC10366134 36543944

[3] Zhou Y DiSclafani M Jeang L Shah AA. Open globe injuries: review of evaluation, management, and surgical pearls. Clin Ophthalmol. (2022) 16:2545–59. doi: 10.2147/OPTH.S372011 PMC9379121 35983163

[4] Stryjewski TP Andreoli CM Eliott D. Retinal detachment after open globe injury. Ophthalmology. (2014) 121:327–33. doi: 10.1016/j.ophtha.2013.06.045 PMC3867520 24011994

[5] Ung C Stryjewski TP Eliott D. Indications, findings, and outcomes of pars plana vitrectomy after open globe injury. Ophthalmol Retin. (2020) 4:216–23. doi: 10.1016/j.oret.2019.09.003 31732470

[6] Li KX Durrani AF Zhou Y Zhao PY Tannen BL Mian SI. Outcomes of penetrating keratoplasty after open globe injury. Cornea. (2022) 41:1345–52. doi: 10.1097/ICO.0000000000002918 PMC9555754 34759204

[7] Osman EA. Glaucoma after open globe injury. Saudi J Ophthalmol Off J Saudi Ophthalmol Soc. (2015) 29:222–4. doi: 10.1016/j.sjopt.2014.10.006 PMC4487842 26155083

[8] Tabatabaei SA Rajabi MB Tabatabaei SM Soleimani M Rahimi F Yaseri M. Early versus late traumatic cataract surgery and intraocular lens implantation. Eye (Lond). (2017) 31:1199–204. doi: 10.1038/eye.2017.57 PMC5558225 28409771

[9] Chuang L-H Lai C-C. Secondary intraocular lens implantation of traumatic cataract in open-globe injury. Can J Ophthalmol. (2005) 40:454–9. doi: 10.1016/S0008-4182(05)80005-5 16116509

[10] Thomas J Armstrong G. Use of Yamane technique for secondary intraocular lens implantation following open globe injury. BMJ Case Rep. (2023) 16:e255995. doi: 10.1136/bcr-2023-255995 PMC10668182 37989326

[11] Andreoli MT Andreoli CM. Surgical rehabilitation of the open globe injury patient. Am J Ophthalmol. (2012) 153:856–60. doi: 10.1016/j.ajo.2011.10.013 22265150

[12] Fernandez EO Miller HM Pham VQ Fleischman D. Comparison of time-to-surgery and outcomes in transferred vs non-transferred open globe injuries. Clin Ophthalmol. (2022) 16:2733–42. doi: 10.2147/OPTH.S378049 PMC9416327 36035239

[13] Agrawal R Rao G Naigaonkar R Ou X Desai S. Prognostic factors for vision outcome after surgical repair of open globe injuries. Indian J Ophthalmol. (2011) 59:465–70. doi: 10.4103/0301-4738.86314 PMC3214417 22011491

[14] Amro MY. Visual outcomes associated with delay from trauma to surgery for open globe eye injury in Palestine: a retrospective chart review study. Lancet. (2021) 398:S14. doi: 10.1016/S0140-6736(21)01500-2 34227945

[15] Djalali-Talab Y Mazinani B Djalali-Talab Y. Traumatic open globe injury—epidemiology, risk factors and visual outcome at the University Hospital Aachen. Spektrum der Augenheilkd. (2021) 35:75–82. doi: 10.1007/s00717-020-00480-4

[16] Fujikawa A Mohamed YH Kinoshita H Matsumoto M Uematsu M Tsuiki E. Visual outcomes and prognostic factors in open-globe injuries. BMC Ophthalmol. (2018) 18:138. doi: 10.1186/s12886-018-0804-4 29884145 PMC5994054

[17] Puodžiuvienė E Valeišaitė G Žemaitienė R. Clinical characteristics, visual outcomes, and prognostic factors of open globe injuries. Medicina (B Aires). (2021) 57(11):1198. doi: 10.3390/medicina57111198 PMC8618771 34833416

[18] Bleicher ID Tainsh LT Gaier ED Armstrong GW. Outcomes of zone 3 open globe injuries by wound extent: subcategorization of zone 3 injuries segregates visual and anatomic outcomes. Ophthalmology. (2023) 130:379–86. doi: 10.1016/j.ophtha.2022.10.027 PMC10038869 36332844

[19] Batchelor A Lacy M Hunt M Lu R Lee AY Lee CS. Predictors of long-term ophthalmic complications after closed globe injuries using the IRIS® registry (Intelligent research in sight). Ophthalmol Sci. (2023) 3(1):100237. doi: 10.1016/j.xops.2022.100237 36561352 PMC9764252

[20] Kuhn F Morris R Witherspoon CD Heimann K Jeffers JB Treister G. A standardized classification of ocular trauma. Ophthalmology. (1996) 103:240–3. doi: 10.1016/S0161-6420(96)30710-0 8594508

[21] R Core Team. R: A language and environment for statistical computing. A Language and Environment for Statistical Computing. Vienna, Austria (2020). Available online at https://www.R-project.org/.

[22] HJ T. Proper method for calculating average visual acuity. J Refract Surg. (1997) 13:388–91. doi: 10.3928/1081-597X-19970701-16 9268940

[23] Holladay JT. Visual acuity measurements. J Cataract Refract Surg. (2004) 30(2):287–290. doi: 10.1016/j.jcrs.2004.01.014 15030802

[24] Moussa G Bassilious K Mathews N. A novel excel sheet conversion tool from Snellen fraction to LogMAR including ‘counting fingers’, ‘hand movement’, ‘light perception’ and ‘no light perception’ and focused review of literature of low visual acuity reference values. Acta Ophthalmol. (2021) 99:e963–5. doi: 10.1111/aos.14659 33326177

[25] Perez EA Scott NL Russell JF. Improved visual outcomes after severe open-globe injuries associated with perioperative vitreoretinal evaluation. Ophthalmol Retin. (2023) 7:771–8. doi: 10.1016/j.oret.2023.04.015 37148970

[26] Kuhn F Maisiak R Mann L Mester V Morris R Witherspoon CD. The ocular trauma score (OTS). Ophthalmol Clin. (2002) 15:163–5. doi: 10.1016/S0896-1549(02)00007-X 12229231

[27] Durrani AF Li K Zhou Y Toiv A Zhao PYC Huvard M. Risk factors for retinal detachment following open globe injury and outcomes of retinal detachment repair following ocular trauma. Invest Ophthalmol Vis Sci. (2022) 63:4302.

